# Zoonoses and the Aboriginal and Torres Strait Islander population: A One Health scoping review

**DOI:** 10.1371/journal.pgph.0000921

**Published:** 2022-10-12

**Authors:** Tamara Riley, Neil E Anderson, Raymond Lovett, Anna Meredith, Bonny Cumming

**Affiliations:** 1 National Centre for Epidemiology and Population Health, The Australian National University, Canberra, Australian Capital Territory, Australia; 2 The Royal (Dick) School of Veterinary Studies and the Roslin Institute, University of Edinburgh, Roslin, United Kingdom; 3 Melbourne Veterinary School, University of Melbourne, Parkville, Victoria, Australia; 4 Animal Management in Rural and Remote Indigenous Communities (AMRRIC), Darwin, Northern Territory, Australia; Institute of Public Health Bengaluru, INDIA

## Abstract

With limited access to animal health services, and high disease burdens among domesticated animals, Aboriginal and Torres Strait Islander communities in Australia face higher risk of disease including zoonoses. However, we lack understanding of the contribution of often preventable zoonoses to the health of these communities, which would enable us to enhance public health strategies and improve health outcomes. We conducted a scoping review to identify the current state of evidence on zoonoses in the Aboriginal and Torres Strait Islander population. We examined the size, scope and characteristics of the evidence base and analysed the zoonoses detected in the studies within a One Health framework. We identified 18 studies that detected 22 zoonotic pathogens in animals, people, and the environment, with most studies detecting pathogens in a single One Health sector and no studies investigating pathogens in all three sectors. Findings indicate that despite the strong conceptual foundations of One Health throughout the evidence base, evidence is lacking in application of this concept. There is a need to undertake further research that prioritises Aboriginal and Torres Strait Islander leadership, considers the contribution of human, animal and environmental health factors, and investigates the prevalence and impact of zoonoses in communities through a One Health approach.

## 1. Introduction

Zoonotic pathogens are a global concern, particularly where animals and humans live closely together. They can be transmitted between animals and people via airborne, vector, direct or indirect contact routes including food borne, water borne and soil borne transmission [1]. Many human pathogens, including emerging infectious diseases, are zoonotic and have originated from animals, particularly wildlife [2]. Endemic diseases (including neglected zoonotic diseases) are of particular concern for low-socioeconomic communities, many of which have a high Indigenous population and are at higher risk of zoonotic disease [3].

The World Organisation for Animal Health (OIE) suggests that controlling zoonotic pathogens in animals is an effective way of protecting people with a One Health approach the optimal method of managing emerging and endemic zoonoses [4]. One Health can be defined as ‘the health of humans, domestic and wild animals, plants, and the wider environment (including ecosystems) are closely linked and inter-dependent’ [5]. One Health is an interdisciplinary approach to health that can result in timely and effective responses and communication, accurate decision making, accountability to other sectors, and shared roles, responsibilities and resources [6,7]. This concept also aligns with Aboriginal and Torres Strait Islander cultural and community contexts that recognise holistic approaches to health and cultural knowledges surrounding the interconnectedness of animals, people, and the environment [8].

In line with the One Health approach, the development of joint health systems, including data sharing and integrated surveillance systems, to improve the management of zoonotic disease across animal and human health sectors is recommended [3]. Centers for Disease Control and Prevention also recognises the importance of multisectoral collaboration in the surveillance, prevention and control of zoonoses including laboratory capacity, outbreak response, and shared management [9]. Within their One Health Zoonotic Disease Prioritisation tool they consider diseases that heavily impact on people as priorities noting that disease rates can vary by population, access to treatment and data availability [9,10].

While the Australian Government has prioritised improvements in Aboriginal and Torres Strait Islander health [11], there continue to be inequities in health outcomes and access to health care for people and animals [12]. This can lead to unmanaged animal populations, reduced access to preventative medicines, and increased risk of zoonotic disease [13,14]. Animals also have the potential to harbour and spread exotic zoonotic pathogens with the north of Australia particularly at risk of incursion due to the vast coastline, remoteness of communities, and the movement of animals and people from neighbouring countries where pathogens are present [15]. Community animal health programs, including surveillance activities, can be beneficial in addressing this risk by increasing the use of preventative medicines, managing animal populations, and increasing awareness of zoonoses [15,16].

Although the management of zoonoses is recognised internationally as important, they are also among the most under-diagnosed diseases in humans with the full burden of disease poorly understood [3]. As a result, the impact of zoonoses on Aboriginal and Torres Strait Islander health and wellbeing remains largely undefined [17]. This review aimed to understand the size, scope and characteristics of the evidence base on zoonoses within the Aboriginal and Torres Strait Islander population in Australia. The findings will be used to further understand priority zoonoses for the population, offer recommendations on applying a One Health approach to the management of zoonoses and inform the development of a community One Health model.

## 2. Methods

### 2.1 Study design

This review was undertaken by an Aboriginal-led research team considering an Australian Indigenous-focused research methodology recognising the importance of prioritizing Indigenous voices and enacting Indigenous leadership within health research [18,19]. A scoping review method was used to examine the body of literature, identify and clarify key concepts, identify knowledge gaps, and inform future research [20]. This allowed us to examine and summarise the size, scope and characteristics of the evidence, and analyse the zoonoses detected in the studies within a One Health framework [21]. Reporting followed the PRISMA extension for scoping reviews checklist (PRISMA-ScR) to ensure all relevant sections were included [22].

### 2.2 Definitions

For this study we used the following zoonoses definition: ‘Infectious diseases that can be spread between animals and humans, and can be spread by food, water, fomites or vectors’ [6, pg. 2]. Therefore we concentrated on pathogens that are commonly transmitted between animals and humans, and have excluded those that have a zoonotic origin but are maintained through human-to-human transmission. We defined the three One Health sectors as follows;

- ’animal’ referred to domestic animals;
- ’human’ referred to people;
- ’environment’ referred to ecosystems including the physical environment, plants, wildlife, and invertebrates that live within an ecosystem.

Studies were assessed for an Indigenous viewpoint which involved the contribution and leadership of Aboriginal and Torres Strait Islander peoples within the research [23], with studies assessed as not having this if it was not explicitly noted. This is appropriate when assessing Indigenous research as methodologies should involve Indigenous leadership and privilege Indigenous voices to allow community priorities to be represented [18,19].

### 2.3 Search strategy

A protocol, including the search terms, was developed in consultation with co-authors to detail the search strategy and reporting considerations. The inclusion criteria included:

- Published peer reviewed research;
- Published between 2000–2021;
- Written in English;
- Full text available;
- Australian Aboriginal and Torres Strait Islander focus;
- Detects a zoonotic pathogen in animals, people and/or the environment.

We conducted the search in August 2021 using public health and university search engines, and an Indigenous specific resource including PubMed, Web of Science, Australian Indigenous HealthInfonet, and the ANU Library Supersearch. The search terms are presented in Table 1.

**Table 1:** Search terms.

Population search terms	Indigenous OR “First Nation*” OR “First People*” OR Aboriginal OR “Torres Strait Islander” OR “Aboriginal and Torres Strait Islander”
Location search terms	Australia OR Australian
Topic search terms	Zoonoses OR zoonosis OR zoonotic

* Includes the plural version of the word in the search.

Records of search results were kept in Excel with references stored in Endnote. Records were sorted by removing duplicates, those that did not have full text, and those that were not original peer-reviewed research articles. Articles were then assessed for inclusion by screening titles and abstracts against the inclusion criteria by three reviewers (TR, BC and NA). Any disagreements were discussed until consensus was achieved.

Data were then extracted from the papers by two reviewers (TR and BC) with each paper reviewed by one reviewer independently. Data were summarised to collect the key points and characteristics extracted included study details, One Health sector, zoonotic pathogen, and gaps and recommendations. Data were grouped by One Health sector (animal, human, and environment), location (state and remoteness), pathogen group (helminths, bacteria, virus, protozoa, and ectoparasites), and Indigenous viewpoint.

We analysed the scope and size of the evidence base and reported on the number of relevant articles from 2000–2021 in an inclusion flow chart and graph. We summarised the characteristics of the evidence base including Indigenous viewpoint, location, One Health sector and species, and displayed results in a Venn diagram. We then analysed the zoonotic pathogens detected and reported them by pathogen group, organism name, number of studies that detected each pathogen, species and location detected, and presented this in a table and pie chart. We also reported which pathogens were investigated but not detected within the evidence base.

## 3. Results

### 3.1. Scope and size of the evidence base

**Fig 1:**
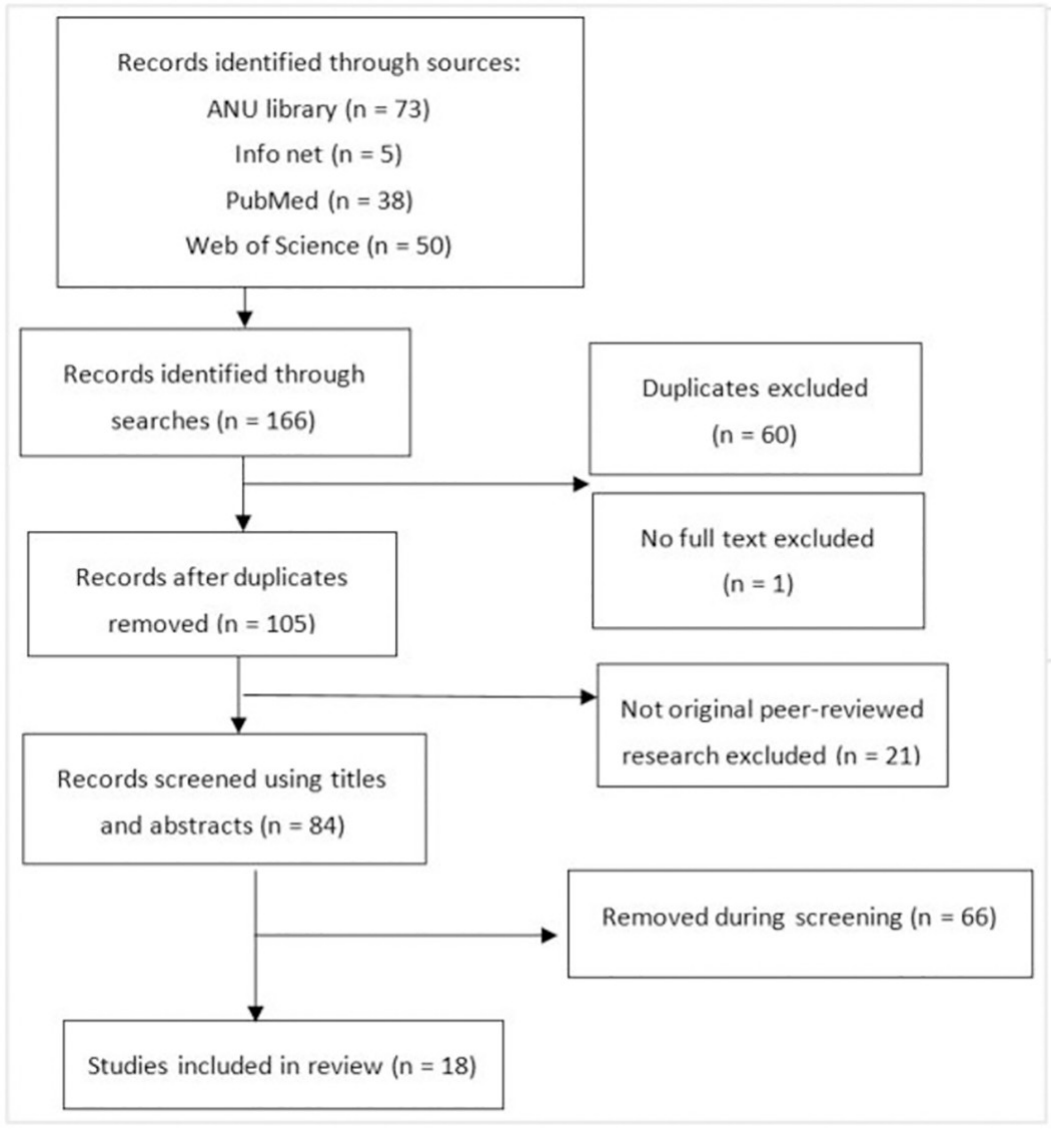
Inclusion and exclusion flow chart.

**Fig 2:**
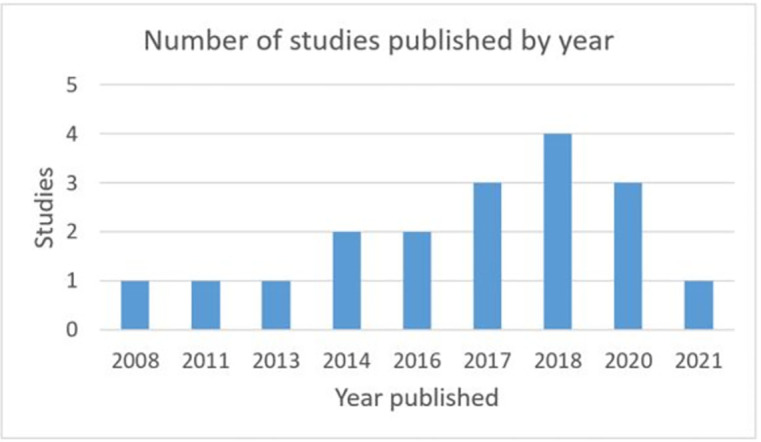
Number of studies published by year.

This review involved the analysis of 18 studies that focused on detecting zoonoses in the Aboriginal and Torres Strait Islander population in Australia (Fig 1). The studies were published from 2008 to 2021, with studies more common from 2014 onwards (Fig 2).

### 3.2. Characteristics of the evidence base

**Fig 3:**
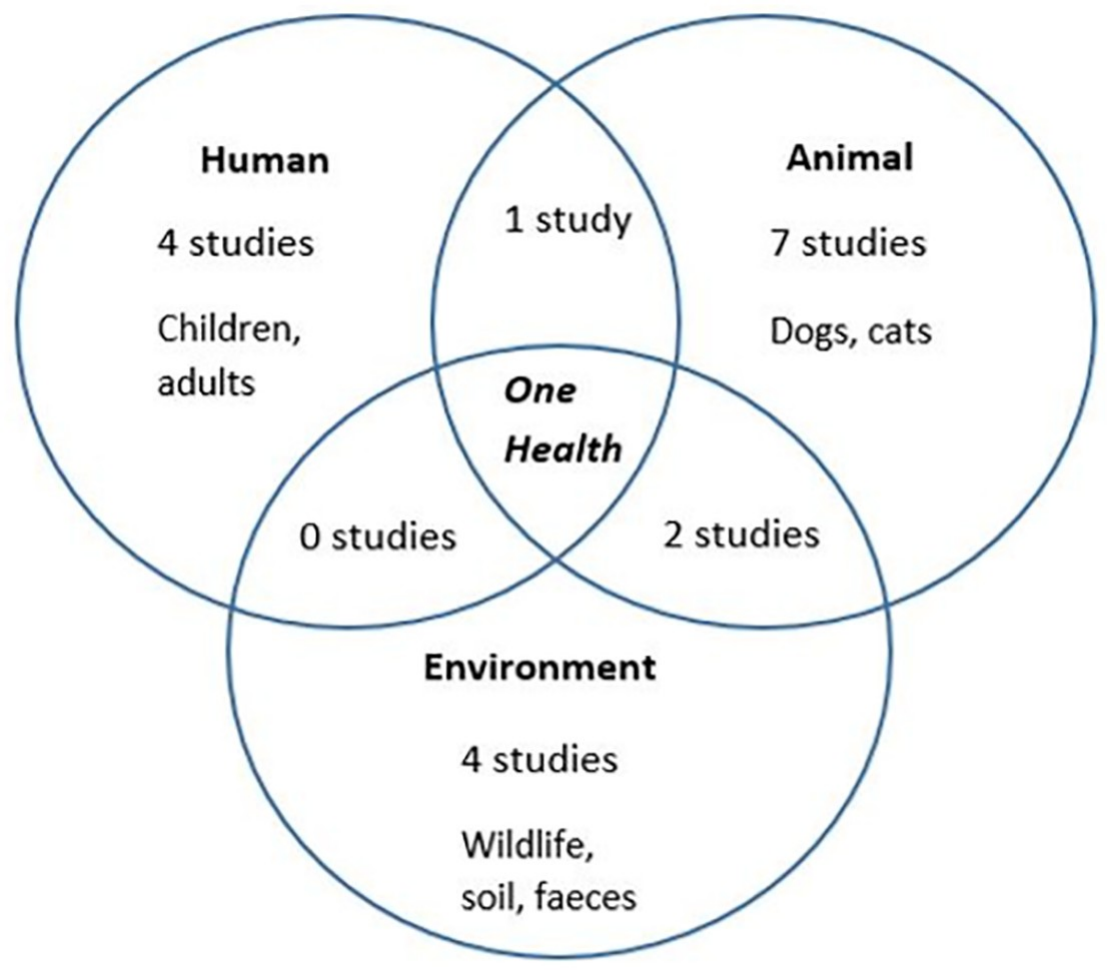
One health sector and species investigated in the evidence base.

There were limited Indigenous viewpoints noted throughout the studies with only four studies meeting this criterion. The majority (n = 15) of studies involved investigation in one sector (animals (n = 7), humans (n = 4), and the environment (n = 4)), three studies looked at two sectors (environment and animals, or animals and people) and no studies looked at all three One Health sectors. Zoonotic pathogens were detected in the following species; dogs (n = 9), dogs and cats (n = 1), humans (n = 5 with three in children and two in adults), and wildlife (n = 4 including dingoes, buffaloes, reptiles and native mammals). Pathogens were also detected in environmental samples including a soil sample (n = 1) and an environmental faecal sample (n = 1 with species unspecified) (Fig 3).

The majority of studies (n = 12) were based in the north of Australia in one state or territory (four in Far North Queensland, five in the Northern Territory, and three in Western Australia). Three studies involved multiple study sites across different states (two in the Northern Territory and western New South Wales, and one in the Northern Territory and Western Australia) and one study was based in western New South Wales only. Two studies were described as based in north and central Australia, however, the location was not specified. At least half of the studies (n = 9) were based in remote communities.

### 3.3. Zoonoses reported

**Fig 4:**
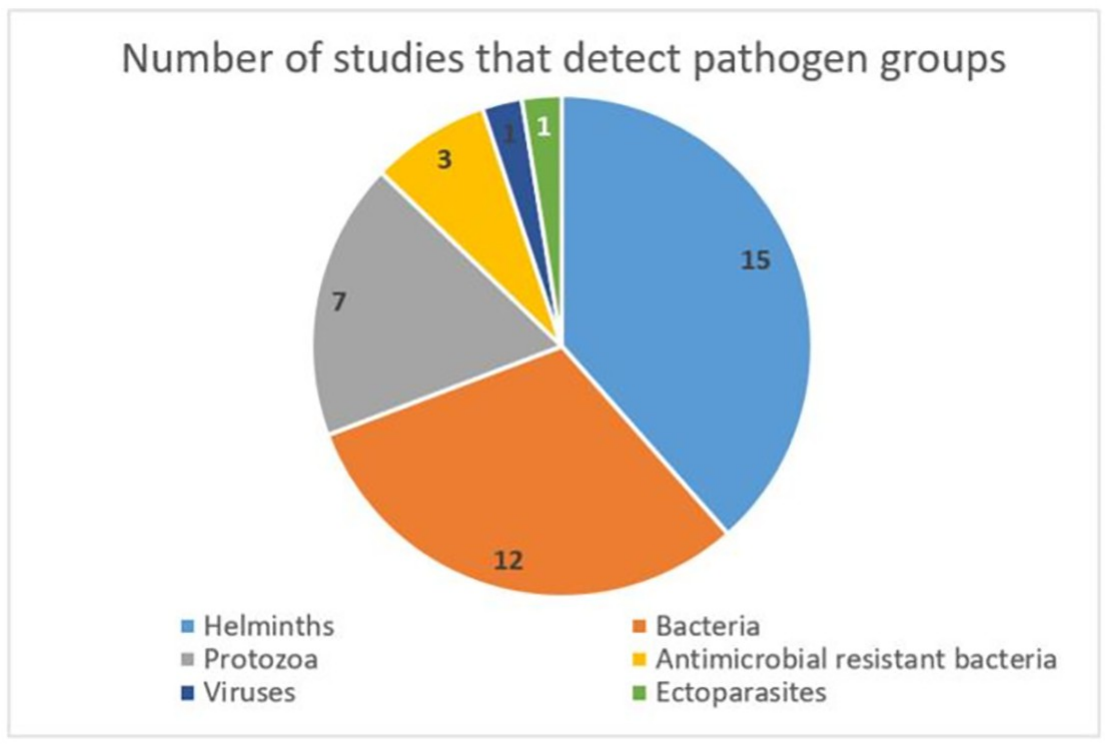
Number of studies that detect pathogen groups.

Within the evidence base 22 zoonotic pathogens were detected. The most common pathogen group was helminths (n = 15) followed by bacteria (n = 12), protozoa (n = 7), antimicrobial resistant bacteria (n = 3), viruses (n = 1) and ectoparasites (n = 1) (Fig 4).

Some pathogens were detected across multiple studies (two pathogens were detected in four separate studies, two pathogens in three studies, and seven pathogens in two studies) with the rest detected in only one study (n = 11) (Table 2). Some studies sampled and tested for, but did not detect, zoonotic pathogens including *Echinococcus granulosus*, *Brucella suis*, *Bartonella vinsonii* and *Bartonella koehlerae*, methicillin-resistant *Staphylococcus pseudintermedius*, *Ancylostoma braziliense*, and *Uncinaria stenocephala*.

**Table 2:** Summary of zoonotic pathogens in the evidence base.

Pathogen group and total studies	Organism name	Number of studies detecting pathogen	Location pathogens detected	Species pathogens detected in	References
Parasites (helminths) Total = 15	*Ancylostoma caninum* (hookworm)	4	Remote north and central Australia; Remote Kimberley Western Australia; Far North Queensland	Dogs Wildlife	[24–27]
	*Dirofilaria immitis* (heartworm)	3	Remote desert, island, rural communities; Far North Queensland	Dogs Wildlife	[26,28,29]
	*Strongyloides stercoralis* (roundworm)	2	Remote north and central Australia; Central Australia	Dogs People	[24,30]
	*Ancylostoma ceylanicum* (hookworm)	2	Far North Queensland	Dogs Wildlife	[25,26]
	*Trichuris vulpis* (whipworm)	1	Far North Queensland	Dogs Wildlife	[26]
	*Toxocara canis* (roundworm)	1	Far North Queensland	Dogs Wildlife	[26]
	*Dipylidium caninum* (tapeworm)	1	Far North Queensland	Dogs Wildlife	[26]
	*Spirometra erinacei* (tapeworm)	1	Far North Queensland	Dogs Wildlife	[26]
Bacteria Total = 12	*Salmonella* spp.	3	Remote desert, island, rural communities; Central Australia	Dogs Wildlife People	[28,30,31]
	*Coxiella burnetti*	2	Central Australia and Moree New South Wales; Northern Territory and north west New South Wales	Dogs	[32,33]
	*Anaplasma* spp. (including *Anaplasma platys*)	2	Remote desert, island, rural communities; Central Australia and Moree New South Wales	Dogs	[28,32]
	*Rickettsia* spp. (including *Rickettsia honei*, *Rickettsia gravesii*, *Rickettsia australis*, *Rickettsia felis*, *Rickettsia typhi*)	2	North west Western Australia; Maningrida, Northern Territory	Dogs	[34,35]
	*Campylobacter jejuni*	1	Central Australia	People	[30]
	*Bartonella henselae*	1	Darwin Northern Territory	People	[36]
	*Streptococcus dysgalactiae*	1	Yarrabah Queensland	Dogs People	[37]
Parasites (protozoa) Total = 7	*Giardia* spp. (including *Giardia duodenalis*)	4	Remote desert, island, rural communities; Remote Kimberley Western Australia; South Arnhem land and Roper Gulf Northern Territory; Central Australia	Dogs Wildlife People	[27,28,30,38]
	*Cryptosporidium parvum*	2	Western Australia; Central Australia	People	[30,39]
	*Cryptosporidium ryanae*	1	South Arnhem land and Roper Gulf Northern Territory	Wildlife	[38]
Antimicrobial resistant bacteria Total = 3	Methicillin resistant *Staphylococcus aureus*	2	Western New South Wales; Remote Kimberley Western Australia	Dogs	[27,40]
	Extended spectrum cephalosporin resistant *E*.*coli*	1	Remote Kimberley Western Australia	Dogs	[27]
Virus Total = 1	Group A *Rotavirus*	1	Northern Territory	People	[41]
Ectoparasites Total = 1	*Sarcoptes scabei*	1	Far North Queensland	Dogs Wildlife	[26]

## 4. Discussion

### 4.1 Summary of findings

Overall, we found a limited number of studies that detected zoonoses within the Aboriginal and Torres Strait Islander population, with studies more common from 2014 onwards highlighting the emerging nature and growth of this area of research. Within the evidence base, there were 18 studies with 22 zoonotic pathogens reported in animals, people and the environment. The most common pathogen group was helminths, followed by bacteria, protozoa, antimicrobial resistant bacteria, viruses and ectoparasites.

The majority of studies detected pathogens within a single One Health sector, most commonly in animals, with a few studies detecting pathogens in people and the environment. It was not common for studies to involve two sectors and no studies involved all three One Health sectors. Investigating zoonotic pathogens in two or more sectors is more appropriate, as it will build on our understanding of the human-animal-environment health dynamic and help to apply genuine One Health approaches [42]. Many zoonoses also involve environmental exposures, however, majority of studies did not involve an environmental component, limiting our understanding of this sectors contribution. Most studies did not describe an Indigenous viewpoint and there is a need for greater involvement of Aboriginal and Torres Strait Islander peoples to incorporate Indigenous voices in the evidence base.

The most common animal species investigated was the domestic dog, with dogs kept as a common pet in communities [43], with the role of cats, livestock, and environmental exposures (including wildlife) in zoonotic disease transmission needing further consideration [44]. The majority of studies were based in north and central Australia, and in remote settings, with two studies based in western New South Wales. There are gaps in knowledge regarding rural communities and those in southern Australia, however, the studies identified did not comprehensively investigate zoonoses in communities therefore, knowledge gaps exist nationally.

Many of the pathogens detected can have a broad range of pathological effects in people and can be difficult to distinguish clinically, leading to underdiagnoses [3]. The pathogens detected in the evidence base can commonly cause problems in multiple human body systems including gastrointestinal, lymphatic, skin, neurological, respiratory, and ocular, with the potential to lead to severe disease. However, it was not possible to assess the contribution of the pathogens to the human burden of disease as the impact on human health was not commonly explored. Although the presence of a pathogen does not equate to the presence of zoonotic transmission, in the limited number of studies that involved diagnosis in people children were commonly involved, which is unsurprising as many of the parasitic zoonoses detected commonly present in children.

Overall, the pathogens detected were reported in one-to-four studies and the majority have not been widely investigated. While these findings have improved our understanding of zoonoses in the Aboriginal and Torres Strait Islander population, the evidence base has not comprehensively investigated the breadth of zoonotic pathogens in communities.

### 4.2 Strengths and limitations

To our knowledge, this is the first study to undertake a zoonoses scoping review using a One Health framework in the Aboriginal and Torres Strait Islander population. Strengths of this study included an Aboriginal-led, multi-disciplinary team, investigating an area of importance for public health with multiple authors assessing the articles for inclusion. We used an Australian Indigenous-focused methodology and a One Health framework to undertake this research. Our findings contribute to the evidence base and inform our understanding of zoonoses in the Aboriginal and Torres Strait Islander population.

The focus on Indigenous research was appropriate due to the study design, however, we recognise this excluded some national and community studies that did not focus on Aboriginal and Torres Strait Islander populations. The review process included three people assessing if an article met the inclusion criteria, however, the data extraction and summary process did not use an interrater process and is therefore a limitation of this review. Inclusion criteria included the detection of a zoonotic pathogen therefore, exotic zoonoses were unlikely to be reported. For example, studies based on rabies were excluded as the pathogen was not detected, however, rabies is relevant for remote communities in the north of Australia due to the risk of incursion and subsequent zoonotic transmission [15,45].

Studies of diseases that have a zoonotic origin but are maintained by human-to-human transmission were also excluded as animals no longer play a key role in transmission and risk of disease. This included diseases such as Covid-19 which is an important disease globally and has been hypothesised to have a zoonotic origin, however, does not commonly transmit between animals and people [46]. The evidence base did not explore the zoonoses prevention and control programs in place, or only discussed programs that exist in one particular sector. Some pathogens were also investigated but not detected, however, as this review did not include a critical analysis of the studies it is not possible to determine whether the lack of detection is meaningful.

Studies that investigate zoonoses in people may have been limited due to inconsistent use of zoonotic terms in the human health sector. We are aware of studies investigating zoonotic pathogens in people that do not characterise these pathogens as zoonotic, and therefore were not identified by the search strategy. This inconsistent use of terminology may be due to lack of awareness and understanding of zoonoses in the human health sector [47], and scientific debate about the zoonotic potential of a pathogen (e.g. *Sarcoptes scabei* [48]).

### 4.3 Implications

Evidence supports controlling zoonotic pathogens in animals as an effective way of protecting people, with a One Health approach optimal [4]. This approach requires strengthening partnerships between animal, human and environmental health sectors for the prevention and management of zoonoses [5,6]. However, a lack of awareness in other sectors has led to the animal sector commonly taking on the responsibility of managing zoonoses. Due to the role of the environment in disease transmission and the ongoing benefits to human health, a joint One Health approach is warranted particularly in low-resourced settings [49,50]. For a One Health approach to be adopted in Aboriginal and Torres Strait Islander populations, more contribution from the human and environmental health sectors is required, as well as consideration of social and cultural determinants of health.

Some of the pathogens detected in the evidence base are of public health importance such as antimicrobial resistant bacteria, with community associated methicillin resistant *Staphylococcus aureus* disproportionately affecting Aboriginal people [27,40]. The prevalence of *Strongyloides stercoralis* is also disproportionately high in Aboriginal communities in Australia [24,30]. *Strongyloides* is a neglected tropical disease, which refers to diseases prevalent in tropical areas that mainly affect low-resourced communities [51]. While strongyloidiasis is not currently nationally notifiable, Strongyloides Australia are leading a push to have this changed [52]. *Coxiella burnetti*, the causative agent of Q Fever, was also identified and is a nationally notifiable disease in people. However, this pathogen is not nationally notifiable in animals and has public health implications in communities where *Coxiella burnetti* has been detected in pet dogs and cats [33,53]. There are also some known zoonotic pathogens that are of public health importance that were not identified in the evidence base (such as *Toxoplasma* and Hendra virus).

There is a need for consistency in zoonoses messaging, case definitions and database management between sectors to effectively manage zoonoses [54,55]. However, Australia does not currently have adequate mechanisms in place to support this with siloed approaches and limited communication between sectors [56]. This leads to challenges in the prevention and control of disease and does not account for the One Health factors that contribute to health. To address this, the development of joint One Health systems is recommended, examples of which can be seen internationally such as in Europe where integrated approaches to monitoring antimicrobial resistance in animals and humans has been implemented [57].

Many of the pathogens identified in the evidence base are transmitted through direct transmission and can be prevented through adequate facilities and infrastructure (housing, sanitation systems, waste management), and preventative health programs (preventative medicines, education and parasite control). The risk of disease can also be related to environmental exposures such as water and food source, and access to wild and feral animals [58]. However, many communities face inadequate facilities regarding environmental management, housing and sanitation, and effective health care leading to an increased risk of disease [59]. One Health approaches can address these inequities by improving access to veterinary programs, human health care, and environmental management, assisting in the prevention of zoonoses.

### 4.4 Future research

There is a need to further investigate zoonoses in the Aboriginal and Torres Strait Islander population through collaboration with multiple health sectors to understand the full impact of zoonotic disease, including epidemiological research to investigate transmission pathways and inform control measures. However, there are complexities when undertaking research in multiple sectors with additional ethics, community approvals, data collection and diagnostic coordination needed to achieve this. Due to the importance of zoonotic pathogens to public health, the impact of zoonoses on human health should be further investigated. Future reviews should consider refining the search strategy to capture human health studies that do not use zoonotic terminology, with multiple authors assessing the zoonotic potential of pathogens. Research should also explore prevention and control programs in place that may influence their findings and consider evaluating the effectiveness of such programs.

To improve the management of zoonoses in communities an Aboriginal and Torres Strait Islander led One Health approach could be beneficial. There is also a need to incorporate Indigenous voices within the evidence base to improve the inclusion of Indigenous viewpoints within research. This is supported by the need for transdisciplinary approaches to community based One Health research with genuine engagement required to emphasise community priorities and achieve sustainable outcomes [60]. This involves community leadership, data sharing, improved collaboration between health sectors, and shared responsibilities to improve health outcomes [8].

This review is the initial step in understanding the impact of zoonoses within the Aboriginal and Torres Strait Islander population and will be built on through future work including an analysis of reported zoonoses in the human health focused National Notifiable Diseases Surveillance System (NNDSS). This approach will allow the comparison of endemic zoonoses highlighted in the evidence base alongside those identified as national public health priorities within the NNDSS. This will enhance our understanding of zoonoses, inform the management of zoonoses, and assist with the development of a community One Health model.

## 5. Conclusion

Within Australia there is limited research undertaken on zoonoses within the Aboriginal and Torres Strait Islander population. As a result, we are yet to understand the full impact of zoonoses on Aboriginal and Torres Strait Islander health and wellbeing. This review identified the current state of evidence on zoonoses in this population using a One Health framework. In the last ten years there has been an increase in studies reporting zoonoses related to Aboriginal and Torres Strait Islander people, however, they are focused mostly on one sector of One Health with no studies focused on all three sectors. Despite the strong conceptual foundations of One Health evidence is lacking in application of this concept in Australia. More evidence on zoonoses through Aboriginal and Torres Strait Islander led and community-driven research, including trends across time and between sectors, is required to improve awareness and identify priorities.

## Supporting information

S1 Checklist (PDF)
